# GS-9219/VDC-1101 - a prodrug of the acyclic nucleotide PMEG has antitumor activity inspontaneous canine multiple myeloma

**DOI:** 10.1186/1746-6148-10-30

**Published:** 2014-01-25

**Authors:** Douglas H Thamm, David M Vail, Ilene D Kurzman, Darius Babusis, Adrian S Ray, Noel Sousa-Powers, Daniel B Tumas

**Affiliations:** 1 Flint Animal Cancer Center, Department of Clinical Sciences, College of Veterinary Medicine and Biomedical Sciences, Colorado State University, 300 W. Drake Rd, Fort Collins, CO 80523-1620, USA; 2 University of Colorado Comprehensive Cancer Center, Denver, CO USA; 3 Barbara A. Suran Comparative Oncology Research Institute, School of Veterinary Medicine and the Carbone Comprehensive Cancer Center, University of Wisconsin-Madison, 2015 Linden Drive, Madison, WI 53706, USA; 4 Departments of Drug Metabolism and Drug Safety Evaluation, Gilead Sciences, Inc, 333 Lakeside Drive, Foster City, CA 94404, USA

**Keywords:** Dog, Plasma cell, Chemotherapy, Guanine

## Abstract

**Background:**

Multiple myeloma (MM) is an important human and canine cancer for which novel therapies remain necessary. VDC-1101 (formerly GS-9219), a novel double prodrug of the anti-proliferative nucleotide analog 9-(2-phosphonylmethoxyethyl) guanine (PMEG), possesses potent cytotoxic activity *in vitro* in human lymphoblasts and leukemia cell lines and *in vivo* in spontaneous canine lymphoma. Given the similarity in lineage between lymphoma and MM, we hypothesized that VDC-1101 would be active against MM.

**Results:**

We evaluated the *in vitro* antiproliferative effects of VDC-1101 against 3 human MM cell lines, and we performed a phase-II clinical trial in 14 dogs with spontaneous MM. Each dog was treated with a maximum of 6 doses of VDC-1101 monotherapy over 10–15 weeks. Dose-dependent antiproliferative activity was observed in all evaluated cell lines. Major antitumor responses (reduction of serum paraprotein and resolution of hypercalcemia, peripheral cytopenias and bone marrow plasmacytosis) were observed in 9 of 11 evaluable dogs for a median of 172 days, including a durable stringent complete response (>1047 days) in a dog with melphalan-refractory disease. 2 dogs were euthanized due to presumed pulmonary fibrosis; there were no other dose-limiting toxicities encountered.

**Conclusions:**

In conclusion, VDC-1101 has significant anti-tumor activity at well-tolerated doses in spontaneous canine MM.

## Background

An estimated 1 in 165 people will be diagnosed with multiple myeloma (MM) in their lifetime and 5-year survival rates are only approximately 34% [1,2]. Despite several recent therapeutic advances, patients with MM still have significant unmet medical need, especially those who fail frontline therapy.

VDC-1101, formerly referred to as GS-9219, is a double prodrug of acyclic nucleotide phosphonate 9-(2-phosphonylmethoxyethyl) guanine (PMEG), which was designed to preferentially deliver and accumulate PMEG and its active phosphorylated metabolite, PMEG disphosphate (PMEGpp) in lymphoid cells while avoiding systemic exposure of PMEG [3]. The delivery of PMEG/PMEGpp results in cytotoxicity due to inhibition of nuclear DNA polymerases α, δ and ϵ [4]. PMEG’s clinical utility is limited by poor cellular permeability as well as gastrointestinal and renal toxicity [5-7]. VDC-1101, however, is hydrolyzed intracellularly to 9-(2-phosphonylmethoxyethyl)-N^6^-cyclopropyl-2,6-diaminopurine (cPrPMEDAP), deaminated to PMEG and then rapidly converted to PMEGpp [3]. In normal laboratory dogs, VDC-1101 selectively depletes replicating lymphoid tissues at doses which spare most other tissues, and demonstrates significant antineoplastic activity in dogs with naturally occurring non-Hodgkin’s lymphoma (NHL) [3,8,9]. VDC-1101 is currently under development as a therapeutic agent for canine cancer.

The evaluation of novel agents in dogs with naturally occurring cancers, especially lymphoid neoplasia, can offer significant insight into an agent’s potential for treatment of human cancers; dogs have similar responses to standard chemotherapeutic agents, an intact immune system, and biologic similarity to humans [10-12]. As in people, canine MM is initially responsive to chemotherapy; however, recurrence of drug-resistant disease occurs commonly, with median survival durations of approximately 18 months following melphalan-based chemotherapy. Long-term relapse-free survival is exceedingly rare [12-14].

Given the encouraging antitumor activity observed in dogs with NHL and owing to the similarity in lineage between B-cell NHL and MM, we sought to determine if VDC-1101 possessed *in vitro* antiproliferative activity against human MM-derived cells and clinical activity in naturally occurring canine MM.

## Results

### *In vitro* antiproliferative effects

**Figure 1:**
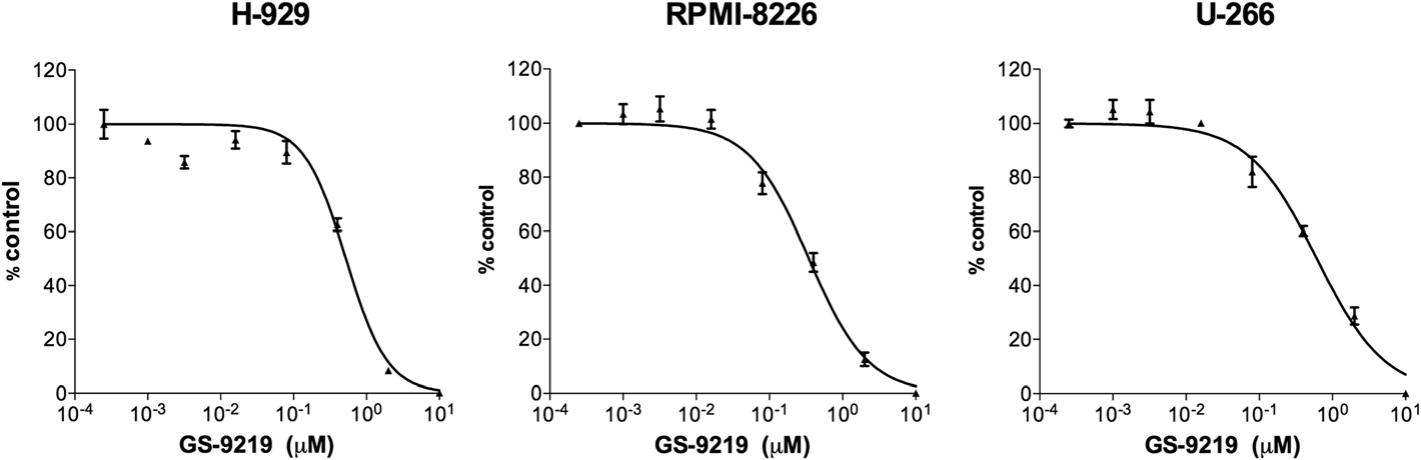
**In vitro antiproliferative effects of VDC-1101 against human multiple myeloma-derived cell lines*.*** Three human myeloma-derived cell lines were incubated with varying concentrations of drug for 5 days, followed by determination of relative viable cell number using a luminescent cell viability assay. VDC-1101 demonstrated dose-dependent inhibition of cell growth in all cell lines examined. Error bars indicate SEM.

VDC-1101 and its bioactive metabolites cPrPMEDAP and PMEG dose-dependently inhibited growth of H-929, RPMI-8226 and U-266 human MM cell lines with IC_50_s between 0.34 and 0.6 μM. The IC_50_s observed with VDC-1101 were roughly the same as those observed with cPrPMEDAP and PMEG, suggesting that MM cells possessed the enzymatic machinery necessary to perform the hydrolysis and deamination of VDC-1101 (Table 1, Figure 1), and are in keeping with the range of *in vitro* IC_50_ reported for other human hematopoietic cell lines [3].

**Table 1:** Antiproliferative activity of VDC-1101 and its metabolites in human multiple myeloma cell lines

	**Calculated IC**_**50 **_**(μM)**		
	**H-929**	**RPMI-8226**	**U-266**
PMEG	1.9 ± 0.6	0.2 ± 0.04	1.0 ± 0.3
cPrPMEDAP	1.8 ± 0.5	0.2 ± 0.06	0.7 ± 0.2
VDC-1101	0.6 ± 0.2	0.34 ± 0.05	0.6 ± 0.2

### *In vivo* treatment and outcome

Fourteen dogs with MM were enrolled prospectively in a clinical trial of VDC-1101 monotherapy, which was administered according to 1 of 3 schedules. Patient and disease characteristics are reported in Table 2. The 4 pretreated dogs all received and subsequently developed progressive disease while receiving melphalan and prednisone, and one had additionally failed VAD (vincristine/doxorubicin/dexamethasone) therapy.

**Table 2:** Patient characteristics

**Median age, years (range)**	**10 (3–14)**
Median weight, kg (range)	28.3 (6.2-40)
Sex	
Male	10
Female	4
Breed	
Labrador retriever	5
Mixed breed	3
Golden retriever	2
Other (1 each)	4
Pre-Treatment	
Yes	4
No	10
Paraprotein	
IgA	10
IgG	2
Light chain only	1
Not determined	1

**Figure 2:**
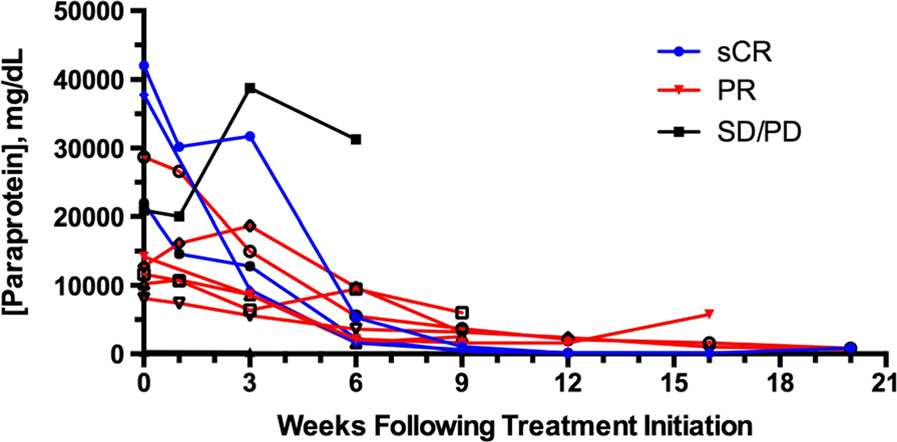
**Serial measurement of paraprotein over time in 11 dogs with spontaneous myeloma receiving VDC-1101 monotherapy*.*** IgA and IgG were measured using radial immunodiffusion. Normal ranges are 40–160 for IgA and 1000–2000 for IgG. One of the dogs experiencing SD/PD as best response is not visible on the graph as pre-treatment IgA concentration was 324 mg/dL.

Response to therapy was assessed utilizing an adaptation of the International Uniform Response Criteria for Multiple Myeloma (Table 3) [15]. Three dogs were not evaluable for response to therapy: 2 dogs were euthanized 1 and 3 days following their first treatment of VDC-1101 owing to development of acute transverse myelopathy (1 definitively due to myeloma-associated vertebral fracture, the other strongly suggested), and 1 dog had light chain disease only and thus paraprotein was not quantifiable. These three dogs were included in outcome analysis on an intent-to-treat basis, and their outcomes treated as events for the purposes of PFI and ST calculations. Of the remaining 11 dogs, 3 experienced stringent complete responses (sCR) and 6 experienced partial responses (PR), for an overall response rate of 82%. Serial measurements of paraprotein in the 11 evaluable dogs are depicted in Figure 2, and pre- and post-treatment bone marrow plasma cell percentages are provided in Table 4.

**Table 3:** Adapted international uniform response criteria for multiple myeloma


**Stringent Complete Response (sCR)**	Normalization of paraprotein
	Disappearance of any soft-tissue plasmacytomas
	<5% plasma cells in bone marrow
	Absence of clonal cells in bone marrow by PARR
**Complete Response (CR)**	Same as above, without clonality criterion
**Partial Response (PR)**	>50% reduction in paraprotein from baseline
	>50% reduction in bone marrow plasma cells
	>50% reduction in soft-tissue plasmacytomas, if present
**Stable Disease (SD)**	Not meeting criteria for CR, PR or PD
**Progressive Disease (PD)**	Increase of 25% from lowest response value in:
	Serum paraprotein
	Bone marrow plasma cell percentage (exceeding 10%)
	Definite development of new bone or soft-tissue lesions
	Definite increase in size of existing bone or soft-tissue lesions
	Development of hypercalcemia not attributable to another cause

**Table 4:** Bone marrow plasma cell percentage before and after VDC-1101 in 3 dogs experiencing stringent complete response

	**Pre-treatment**	**Following final treatment**
**Patient AM**	No marrow elements present	2% well-differentiated plasma cells
**Patient KW**	40-50% atypical plasma cells	<1% morphologically normal plasma cells
**Patient SR**	30% atypical plasma cells	2-5% well-differentiated plasma cells

NB: patient AM’s myeloma diagnosis was made based on hypercalcemia, monoclonal gammopathy and the presence of multiple bony lytic lesions on radiographs.

Dogs presented with a variety of MM-associated hematologic and biochemical abnormalities, most of which resolved or substantially improved in response to VDC-1101 monotherapy (Table 5). The notable exception was azotemia, which tended not to improve.

**Table 5:** Myeloma-Related clinicopathologic abnormalities

	**N**	**Improved**	**Resolved**
**Hyperglobulinemia**	11		8
**Hypoalbuminemia**	10		8
**Leukopenia**	10		9
**Anemia**	8	2	6
**Thrombocytopenia**	8	1	7
**Hypercalcemia**	4	1	3
**Azotemia**	3		
**Elevated liver enzymes**	2		2

5 dogs were considered censored in the PFI analysis owing to euthanasia for unrelated reasons (other neoplasia, owner request despite PR, pre-existing azotemia unresponsive to therapy, n = 3), ongoing CR (n = 1) or loss to follow-up (n = 1). The median follow-up time in censored patients was 72 days. The median overall PFI was 124 days (1 to >1047 days), and the median PFI in patients experiencing PR or sCR was 172 days (95 to >1047 days). The median PFI was not reached in patients experiencing sCR and 130 days in patients experiencing PR; this difference was not statistically significant (*p =* 0.10). There was no statistical difference in response rate or PFI between dogs receiving VDC-1101 once every 21 days and those receiving other schedules (124 vs 172 days, *p* = 0.46). One dog was re-treated with VDC-1101 at the time of relapse (410 days following treatment initiation) and one dog went on to receive additional specific antineoplastic therapy following VDC-1101 therapy (prednisone and melphalan). The overall median ST was 110 days. This number is less than the median PFI owing to differential censorship of some patients in PFI and ST calculations.

**Figure 3:**
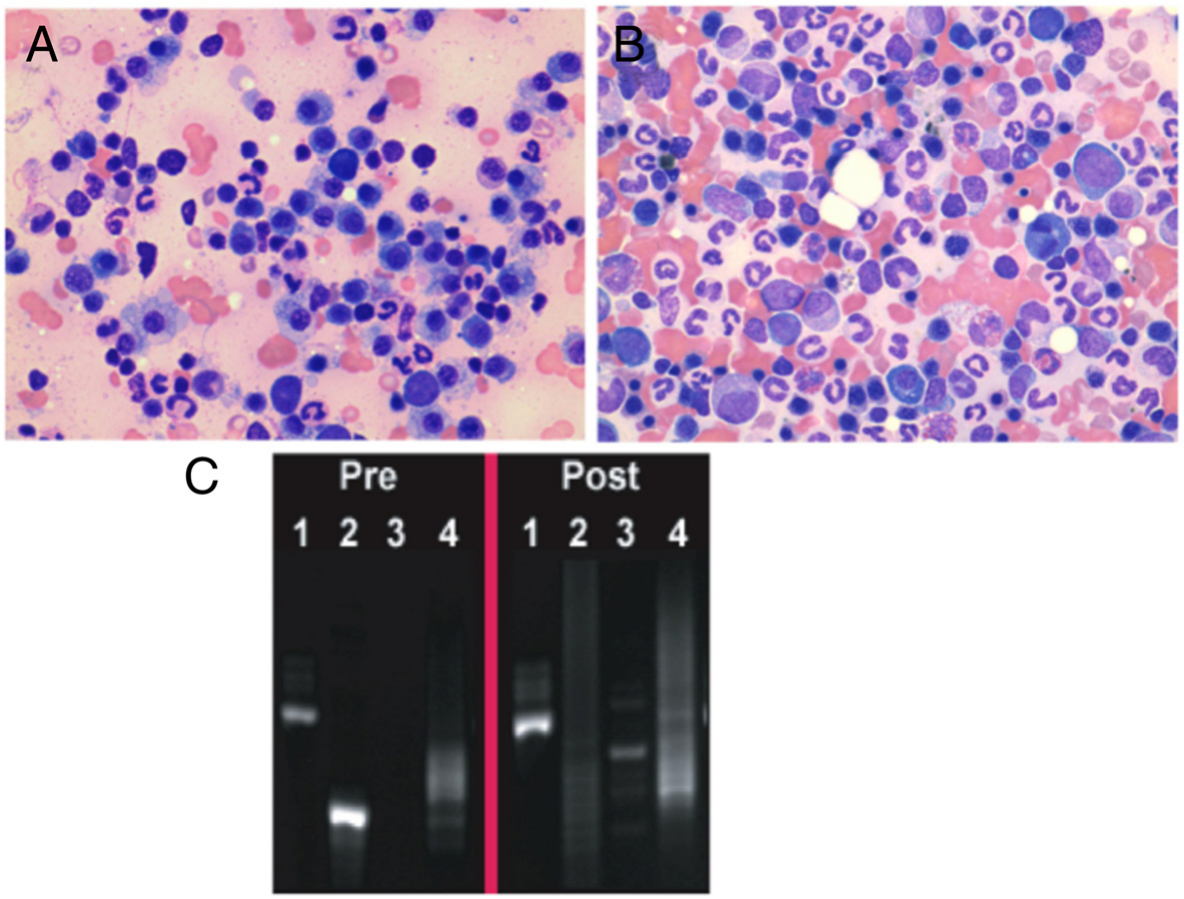
**Bone marrow cytologic and molecular complete response in a dog with spontaneous myeloma receiving VDC-1101 monotherapy.** The photomicrographs above represent pre-treatment **(A)** and post-treatment **(B)** bone marrow cytology from an 8 year old male castrated Labrador retriever with melphalan-refractory myeloma. The pre-treatment sample contained 30% atypical plasma cells, whereas the post-treatment sample contained 2% morphologically normal plasma cells. PCR for antigen receptor rearrangement was performed on bone marrow obtained from this patient prior to and following VDC-1101 **(C)**. 1: Positive control for amplifiable DNA. 2,3: PCR products generated employing 2 distinct sets of immunoglobulin gene primers. 4: PCR products generated employing primers for T cell receptor gene. A solitary band, indicating a monoclonal immunoglobulin gene rearrangement, is present in Lane 2 of the pre-treatment sample. This is replaced with polyclonal immunoglobuin gene rearrangements in Lane 2 of the post-treatment sample, indicating disappearance of the malignant clone.

Of note, 1 dog that had failed previous therapy with prednisone and melphalan experienced a sCR following 5 treatments with VDC-1101 and was euthanized 1047 days following treatment initiation owing to the development of an unrelated neoplasm (apocrine gland carcinoma of the anal sac). There was no gross or histologic evidence of MM on necropsy in this dog. Representative bone marrow photomicrographs and results of PARR from this dog are presented in Figure 3. A second dog remains alive and in sCR >300 days following treatment initiation.

### Safety and adverse events

Adverse events were transient and self-limiting in most dogs and are described in Table 6. The single episode of grade 4 neutropenia occurred after the first dose and was not accompanied by fever. In this dog, significant neutropenia (1000 neutrophils/μL) was present prior to treatment and normalized with continued therapy. Dermatopathy was noted in 6 dogs (2 dogs with grade 1 and 4 dogs with grade 2 severity), and consisted most commonly of focal mild otitis or pruritic and erythematous skin lesions on the dorsum. The skin lesions resolved with standard of care therapy including topical treatments and systemic analgesics and antibiotics as needed. A 1-week dose delay was instituted for one dog for management of dermatopathy. Two dogs developed severe dyspnea 124 and 135 days following treatment initiation, which led to euthanasia owing to respiratory signs. Both of these dogs were experiencing PRs at the time of euthanasia, and pulmonary fibrosis was confirmed on necropsy.

**Table 6:** Adverse events

	**Grade 1**	**Grade 2**	**Grade 3**	**Grade 4**	**Grade 5**
**Hypercalcemia**	2*				
**Hypomagnesemia**	1				
**Hypercholesterolemia**	6				
**Hyperchloremia**	1				
**Increased liver enzymes**	5	2	1		
**Hypokalemia**	3				
**Hyponatremia**	3				
**Hypochloremia**	3				
**Neutropenia**		2	1	1**	
**Increased creatinine**		1			
**Increased BUN**	1				
**Hypophosphatemia**	1				
**Lethargy**	1				
**Anorexia**	1				
**Diarrhea**	1				
**Pulmonary**					2
**Dermatopathy**	2	4			

*Associated with disease progression in 1 patient.

**Neutropenia existed prior to treatment in this patient.

Postmortem examinations were performed in 8 dogs. Extensive infiltration of plasma cells into bone marrow and parenchymatous organs was demonstrated in 6 dogs. One dog demonstrated hepatic fibrosis and 3 had renal changes (combinations of renal fibrosis, glomerulopathy, subacute tubular nephrosis, renal infarct, and chronic interstitial nephritis). The aforementioned diffuse pulmonary fibrosis was noted in 2 dogs, and a third dog had a focal area of lung fibrosis admixed with malignant plasma cells.

## Discussion

Despite advances and development of novel anti-MM agents such as immunomodulators, bisphosphonates and proteasome inhibitors [16], conventional and novel cytotoxic agents likely will continue to play an important role in MM management [17]. VDC-1101, novel prodrug of the guanine nucleotide analog PMEG, has significant antitumor activity and acceptable tolerability in dogs with NHL [3,8,9]. Based on similarities in NHL and MM lineage and similarities between canine and human MM, we investigated the activity of VDC-1101 in dogs with naturally occurring MM.

VDC-1101 exhibited dose-dependent antiproliferative activity against 3 human MM cell lines, with IC_50_ values in the range of those reported against other human lymphoma and leukemia cell lines, supporting the evaluation of VDC-1101 in dogs with MM. Clear and significant antitumor activity was demonstrated with VDC-1101 by clinicopathologic assessment of serum paraproteins, bone marrow cytology, and a sensitive PCR-based assay for detecting clonal immunoglobulin gene rearrangement, as well as normalization or substantial improvement in a variety of MM-associated clinicopathologic abnormalities. Notably, a complete and exceptionally durable sCR was observed in a dog with melphalan-refractory myeloma, which is rarely observed in dogs with MM [13], and a second sCR is ongoing at >300 days. Additionally, MM is generally treated continuously in both dogs and humans. The fact that durable sCRs have been observed and maintained following 5 doses of VDC-1101 without any maintenance therapy is likewise encouraging.

The activity observed in both untreated and melphalan-refractory patients indicates potential utility for VDC-1101 in both induction and rescue chemotherapy settings. Given the lack of any obvious schedule-dependency on response rates or durations, a dosage of 0.82 mg/kg VDC-1101 free base (1.0 mg/kg succinate salt) as a 30-minute IV infusion once every 21 days is recommended for further study.

Acute adverse events were transient and manageable, and consistent with those described in normal dogs and in dogs with NHL treated with GS-9219 [3,9]. Severe pulmonary fibrosis leading to euthanasia was identified in 2 dogs experiencing PR. This phenomenon has been observed previously in a small percentage of dogs with NHL treated with VDC-1101, typically several months following treatment discontinuation [9]. The observance of this adverse event in NHL patients led us to pursue concurrent low-dose prednisone therapy in one patient treated in this study. Future studies will investigate the ability of concurrent corticosteroids to mitigate this serious adverse event, and careful monitoring of thoracic radiographs and assessment for pulmonary signs is warranted.

## Conclusions

In conclusion, VDC-1101 exhibits substantial activity against naïve and refractory MM in dogs, with acceptable toxicity. Pulmonary fibrosis can be observed in some MM patients treated with VDC-1101, and patients should be closely monitored for this adverse effect.

## Methods

### *In vitro* growth inhibition

The U-266, RPMI-8226 and H-929 human MM-derived cell lines were obtained from ATCC (Manassas, VA). Cell culture media containing Glutamax was purchased from Invitrogen (Carlsbad, CA). CellTiter-Glo^®^ was purchased from Promega (Madison, WI). VDC-1101, cPrPMEDAP and PMEG were synthesized by Gilead Sciences.

The U-266 cell line was maintained in RPMI-1620 medium supplemented with 15% fetal bovine serum (FBS). The RPMI-8226 and H-929 cell lines were cultivated in RPMI-1620 containing 10% FBS. In the case of H-929, 2-mercaptoethanol with a final concentration of 0.05 mM was also added. All cells were incubated under standard conditions (37°C, 5% CO_2_, humidified).

Cells (67,000 cells/mL) were distributed into 96-well plates in 150 μL of media/well and incubated overnight. The following day, 5-fold serial dilutions of the tested compounds were prepared and the diluted compounds were added in duplicate in 50 μL/well to the cells. After a 5-day incubation, 100 μL of cell suspension was removed from each well and cell viability was assed using a luminescent cell viability assay (CellTiter-Glo^®^, Promega, Madison, WI) following manufacturer protocol. Values were expressed as a percentage of untreated cells and 50% inhibitory concentration (IC_50_) values were derived by fitting dose–response curves using a sigmoidal dose response equation.

### Patient population

Pet owners presenting to the School of Veterinary Medicine, University of Wisconsin-Madison (UW-SVM) or the Colorado State University Veterinary Medical Center (CSU-VMC) were offered study entry for treatment of their dogs with VDC-1101 under Colorado State University IACUC protocol # 06-100A-03 and University of Wisconsin IACUC protocol # V01287. Signed informed consent was obtained from all owners. Prior to entry, dogs were evaluated by physical examination, complete blood count (CBC), serum biochemistry profile, urinalysis, thoracic radiographs, bone marrow cytology, skeletal survey radiography, and immunoglobulin quantification by radial immunodiffusion as described [18]. Concurrent antineoplastic therapy was not allowed, with the exception of low-dose prednisolone (1 mg/kg PO every other day) in a single dog. Previous cytotoxic chemotherapy was allowable with a 3-week washout prior to enrollment.

### VDC-1101 Administration protocol

In 4 dogs, VDC-1101 was administered by a 30-minute intravenous infusion in 5% dextrose for injection (2 mL/kg) at a dosage of 0.82 mg/kg free base (1.0 mg/kg succinate salt) on weeks 0 and 1, then 0.66-0.82 mg/kg on weeks 4, 7, and 10. In 9 dogs, VDC-1101 was administered as above at a dosage of 0.82 mg/kg every 3 weeks for a maximum of 5 treatments. In 1 dog, VDC-1101 was administered at a dosage of 0.25 mg/kg daily for 5 consecutive days, repeated every 21 days for 5 treatment cycles. The differences in administration protocol were due to the fact that these 3 schedules of administration were being evaluated concurrently in dogs with NHL at the time these patients were treated [9].

### Safety evaluation

Adverse events were graded prospectively according to the Veterinary Cooperative Oncology Group Common Terminology Criteria for Adverse Events v1.0 [19], based on client history, physical examination, CBC, biochemistry profile, and urinalysis. Evaluations were performed pretreatment and at all visits thereafter. If dogs experienced grade 3 or greater adverse events, dose reduction or delay was instituted. Whenever possible, postmortem examinations were performed at the time of death.

### Efficacy evaluation

At each visit including monthly re-checks post-treatment, owner history, physical examination, CBC, serum biochemistry, urinalysis and immunoglobulin quantification were performed. Prior to and 1 month following the final VDC-1101 infusion in dogs experiencing clinical CR, bone marrow was aspirated for cytology and PCR for antigen receptor rearrangement (PARR) for clonality assessment. This was performed as described in 2 cases [20], and using a modification of the published protocol in 1 case, methods for which are given in Additional file 1. Response to therapy was assessed utilizing an adaptation of the International Uniform Response Criteria for Multiple Myeloma (Table 3) [15]. The progression-free interval (PFI) was defined as the interval from treatment initiation to development of progressive disease. Survival time (ST) was defined as the interval from treatment initiation until death. Dogs were censored from analysis if they were in remission at the time of last treatment, lost to follow-up or died of disease other than MM. The PFI and ST were calculated using the Kaplan-Meier product-limit method, which accounts for dogs that were in remission at the time of last follow-up, lost to follow-up or died of disease other than MM and are statistically referred to as censored. Outcome comparisons between groups were made using logrank analysis. All statistical analyses were performed using a commercial software package (Prism v6.0, GraphPad Software, LaJolla, Ca).

## Competing interests

This study was supported by Gilead Sciences, Inc. D. Babusis, A. Ray and D. Tumas are employees and/or shareholders of Gilead Sciences, Inc. D. Thamm and D. Vail are paid consultants for, and D. Thamm is a shareholder in VetDC Inc., the current licensee of VDC-1101 for veterinary use.

## Authors’ contributions

DHT, DV and DBT were collectively responsible for study design. DHT and DV were responsible for the clinical aspects of study performance. DB and AR participated in *in vitro* data collection, analysis and interpretation. NS and IK participated in clinical data collection, analysis and interpretation. DHT was the primary manuscript author, with extensive assistance from DV and DBT. All authors read and approved the final manuscript.

## Supplementary Material

Additional file 1 PARR assay methods. Click here for file
