# Supplementary material for: GS-9219/VDC-1101 - a prodrug of the acyclic nucleotide PMEG has antitumor activity inspontaneous canine multiple myeloma
Source: BMC Vet Res. 2014 Jan 25;10:30. doi: 10.1186/1746-6148-10-30 (PMC3904015; doi:10.1186/1746-6148-10-30)
Supplement: Additional file 1 — PARR assay methods. [file 1746-6148-10-30-S1.docx]

**PARR Assay Methods**

For the PARR assay, DNA was isolated from aspirates using the blood and body fluid protocol with the QIAmp DNA mini kit. The PARR assay to detect rearranged immunoglobulin genes was performed using the following primers, where Rho amplifies the rhodopsin gene (positive control) and the Brd primers are located in conserved regions of immunoglobulin V genes (forward primers) or J genes (reverse primers).

RhoCeF F 56FAM-ACCACCCAGAAGGCTGAAA

RhoCeR R CTGGGAGGGTCATGAAGATG

Brd1 F GGGGAGACCTGGTGAAGCC

Brd2 F TGGAGTCTGGGGGAGACCT

Brd3 R VIC-ACCTGAGGAGACGGTGACC

Brd4 R VIC-TGAGGACACGAAGAGTGAGG

1.2 ul of DNA was added to 25ul of reaction mixture containing Qiagen Multiplex Master Mix, primer and water. Cycling conditions were as follows:

95ºC 15:00, 1 cycle; 94ºC 0:30 64ºC 0:30, decrease temp after cycle 1 by 0.5 ºC every cycle, 72ºC 01:30, 10 cycles; 94ºC 0:30, 59ºC 0:30, 72ºC 01:30 30 cycles.

Single stranded PCR products were analyzed using an ABI 3100 Genetic Analyzer, which allows for 1 base size resolution. The size of the rhodopsin product is 147 bases, and the size range for the immunoglobulin products is 300 – 380 bases.
